# Fetal exposure to dichloroacetic acid and impaired cognitive function in the adulthood

**DOI:** 10.1002/brb3.1801

**Published:** 2020-08-25

**Authors:** Yue Wang, Wenbo Jiang, Qiuying Dong, Yue Zhao, Yingying Chen, Changhao Sun, Guoli Sun

**Affiliations:** ^1^ Department of Nutrition and Food Hygiene College of Public Health Harbin Medical University Harbin P. R. China; ^2^ Department of Experimental Center The First Hospital of Harbin City Harbin P. R. China; ^3^ The first Psychiatric Hospital of Harbin Harbin China

**Keywords:** BDNF, dichloroacetic acid, hippocampus, neuroinflammation, oxidative stress, synaptic plasticity

## Abstract

**Introduction:**

Dichloroacetic acid (DCA), a by‐product of disinfection in drinking water, is a multiple organ carcinogen in humans and animals. Still, little research on its neurotoxicity and its underlying mechanism has not been elucidated.

**Methods:**

Sprague Dawley rats were intragastrically treated with DCA at 10, 30, 90 mg/kg body weight from pregnancy till delivery. At eight weeks of age of pups, we assessed cognitive performance using the standard behavioral tests. And the hippocampus structure and ultrastructure were evaluated using light and electron microscope. The oxidative stress indicators and neuroinflammation factors were measured with the corresponding kits. The mRNA and protein of synaptic factors were detected using RT‐PCR and Western blot.

**Results:**

The results indicated that maternal weight gain and offspring birthweight were not significantly affected by DCA. However, behavioral tests, including morris water maze and step down, showed varying degrees of changes in DCA‐treated pups. Additionally, we found significant differences in hippocampal neurons by histomorphological observation. Biochemical analysis results indicated superoxide dismutase (SOD) and catalase (CAT) activities, as well as reactive oxygen species (ROS), nitric oxide (NO), and reduced glutathione (GSH) levels, were affected by DCA accompanying with DNA damage. Moreover, the results showed that the neuroinflammation factors (TNF‐α, IL‐6, IL‐1β) in DCA treatment groups increased significantly compared with the control pups. And we also found that DCA treatment caused a differential modulation of proteins (BDNF, cAMP‐response element‐binding protein1 (CREB1), p‐CREB1, postsynaptic density‐95 (PSD‐95), synapsin I, p‐synapsin I), and mRNA (BDNF, PSD‐95).

**Conclusions:**

Taken together, these results above showed that oxidative stress, neuroinflammation response, and weakened synaptic plasticity in pups hippocampus induced by fetal exposure to DCA could damage the function of memory and cognition in the adulthood.

## Graphical abstract

There are still no evidence reporting whether DCA could hamper the memory and cognitive function, and it is still largely unknown whether the roots of impaired cognitive function extend back to exposure to DCA during early life.Therefore, we proposed hypothesis that fetal exposure to DCA may cause oxidative stress, inflammatory response, and damaged neurotrophic factors, which could interrelate to affect synaptic plasticity and cognition function in the adulthood.

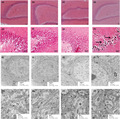

## 1 INTRODUCTION

Importance of the intrauterine environment for the entire life‐course is emphasized by the developmental origin of health and disease hypothesis, and abundant studies have demonstrated that fetal exposure to harmful stimulating factors partially contributes to the development of the central nervous system and risk of schizophrenia in adulthood (Kang et al., 2017; Vohr, Poggi Davis, Wanke, & Krebs, 2017).

Dichloroacetic acid (DCA), a by‐product of the disinfection of drinking water, has been nominated to the National Toxicology Program by the United States Environmental Protection Agency for toxicity and carcinogenicity studies (Wehmas et al., 2017). An increasing amount of rodent studies have confirmed the fact that DCA has been linked etiologically and epidemiologically to several disorders, including spermatotoxicity, immunotoxicity, and carcinogenicity (Cai et al., 2007; Wood et al., 2015). However, there are few studies on the toxicity of DCA to the central nervous systems.

Moser et al. demonstrated DCA could produce neuromuscular toxicity comprised of limb weakness, and deficits in gait and decreased hindlimb grip strength were the earliest indicators of toxicity (Moser, Phillips, & McDaniel, 1999). However, there is still no evidence reporting whether DCA could hamper the memory and cognitive function, and it is still largely unknown whether the roots of impaired cognitive function extend back to exposure to DCA during early life.

The mechanism of DCA‐induced toxicity is considered to be related to mitochondrial depolarization and then induces ROS overproduction (Arany et al., 2013). And nuclear factor‐κB is a crucial transcription factor that regulates the expression of pro‐inflammatory cytokines and inflammatory mediators genes, which is known to exacerbate the underlying pathogenesis of neurotoxicity, which may develop into cognitive impairment (Ye et al., 2015). It is a well‐known neurotrophic factor involved in activity‐dependent synaptic plasticity and seems to protect neurons against different kinds of brain insult (Duman, Deyama, Fogaça, 2019). And it could facilitate synaptic transmission and regulate gene expression through activation of synapsin I and CREB1, which have been implicated in synaptic function underlying learning and memory (Wu, Ying, & Gomez‐Pinilla, 2004). Therefore, we proposed the hypothesis that fetal exposure to DCA may cause oxidative stress, inflammatory response, and damaged neurotrophic factors that could interrelate to affect synaptic plasticity and cognition function in adulthood.

## 2 MATERIALS AND METHODS

### 2.1 Chemicals and reagent

Dichloroacetic acid (MW:128.94; purity:99%) was obtained from Aldrich Chemical Co. (Milwaukee, WI), and the DCA solution was made by dissolving the compound in deionized water and stored in sealed opaque glass.

### 2.2 Animal studies

Forty male and female *SD* rats (aged 4–6 weeks) from Harbin Medical University Laboratory Animal Center (Harbin, China) were housed with a 12 hr light/dark cycle at a constant temperature (22 ± 3℃) and relative humidity (40%–70%). All works were approved by Harbin Medical University Ethics Committee for animal research and conformed to the policy and regulation for the Care and Use of Laboratory Animals approved by the Institutional Animal Care and Use Committee. After a one‐week accommodation period, the rats were then mated, and the females were examined the next morning for vaginal smears. Once showing sperm smears, the rats were regarded as pregnant and were housed individually. The pregnant rats were administered DCA (10, 30 or 90 mg/kg body weight) solution via intragastric injection and weighed twice a week (on Wednesday and Sunday). The drug doses were selected based on the pilot study in our laboratory preliminary experiments and previous reports (El Arem et al., 2017), which would like to be a continuum between no‐effect and substantial‐effect levels. After the treatment period, the dams and infirm pups were killed. At eight weeks of age, following the behavioral tests, the pups were then euthanized. The whole brains were removed and rinsed in glassware filled with precooling isotonic NaCl. The hippocampi were immediately separated and snap‐frozen in liquid nitrogen for subsequent experiments and analysis.

### 2.3 Behavioral tests

#### 2.3.1 Morris water maze

At eight weeks of age, the spatial orientation and memory of the pups were evaluated with the morris maze test, as previously described (Rahman, Rao, & Khan, 2018). This equipment consisted of a round pool (100 cm diameter, 58 cm height) filled with tepid water at a temperature of 22–24℃. A plexiglass platform (40 cm high and 10 cm diameter) was installed about 2 cm below the water surface in the center of the second quadrant during the training session. An automatic tracking system was utilized to monitor swimming activities. The spatial training protocol included training trials to assess acquisition and probe trials to determine the search strategy.

Briefly, section one (training test): Rats were given four training trials each day for four consecutive days using a 60 s intertrial interval. In each trial, rats were put into the water facing the tank wall in one of the four quadrants in a stochastic order each day. The rats remained on the platform for 15 s before removal. The rats that failed to reach the platform within 120 s were guided to it by the experimenter. Each rat was then kept on the terrace for 10 s, and escape latency and times crossing platform site were recorded.

Section two (spatial probe test): The platform was retracted from the pool, and a 2 min probe test was conducted and recorded the time in the target zone for up to 2 min.

#### 2.3.2 Step‐down test

The device consisted of a plastic box (15 cm × 14 cm × 40 cm) with parallel 0.1 cm‐caliber stainless steel bars on the bottom and an elevated rubber terrace (diameter: 5 cm, height: 5 cm). The step‐down tests included training and passive avoidance sessions. During the training session, the rat was placed in the box for 5 min, during which they were permitted to move freely. Then, a 36 V foot shock was given through the bar. The stimulated rat jumped to the terrace to avoid the shock and once jumped down to the grid and was immediately punished by another foot shock. Passive avoidance section was started after 24 hr, the rats were placed on the terrace, and the time it took to step down was recorded as the escape latency, an index of memory retention.

### 2.4 Histomorphology

#### 2.4.1 Optical microscopy

The rats were fixed by rapid heart perfusion with cold saline, followed by 4% paraformaldehyde after liver turning white. After perfusion, the hippocampal tissue was taken off and placed into 4% paraformaldehyde fixative solution for 24 hr. Besides, hippocampal tissue was rinsed with 0.01 mol/L PBS three times, dehydrated with graded alcohol (10%, 20%, and 30%), cleaned again in dimethyl benzene, embedded with paraffin wax and made into 4mm slices and then observed and photographed with an optical microscope (Olympus, Tokyo, Japan) after HE staining.

#### 2.4.2 Electron microscopy

The hippocampal specimen was cut into 1 mm^3^ sized cubes and quickly placed in chilled 3% glutaraldehyde for 48 hr, followed by 0.01 mol/L PBS buffer. And then, it was fixed in 1% osmium solution for 2 hr, rinsed in identical PBS, dehydrated in graded alcohol (10%, 20%, and 30%), and blocked with araldite. The slices stained with uranyl acetate and lead citrate were then observed with an electron microscope.

### 2.5 Measurement of SOD, CAT activity, production of ROS, NO, and reduced GSH level in hippocampus

Measurements of enzymatic activities of SOD, GSH, CAT, ROS, and NO contents in the hippocampus were performed spectrophotometrically using the corresponding kits (Nanjing Jiancheng Bioeng Inst, China) according to the manufacturer's protocols.

### 2.6 Enzyme‐linked immunosorbent assay (ELISA) for BDNF and neuroinflammation factors

The BDNF and neuroinflammation factors (NF‐κB, TNF‐α, IL‐6, and IL‐1β) concentration in the hippocampal tissue was measured using ELISA kits, according to the manufacturer's instructions (Elabscience Biotechnology Co., China). The hippocampal tissue and standard preparations were added to the enzyme‐labeled plates according to the instructions. The plates were sealed and incubated at 37°C for 45 min. The ELISA plates were washed three times with wash buffer (1×), and horseradish‐peroxidase‐conjugated fluid (100 μl) was added to each well and incubated for 30 min at 37°C. The plates were then washed five times, and substrate reagent (3,3′,5,5′‐tetramethylbenzidine) was added to each well and incubated 15 min at 37°C for in the dark. Stop Solution (50 μl) was added to the wells, and the absorbance was then measured at 450 nm in a microplate reader (Bio‐Tek Elx800, Bio‐Tek).

### 2.7 Measurement of DNA damage by the comet assay

The hippocampal tissue was rinsed twice with PBS and broken into pieces to make into each cell suspension and then incubated in trypsin at 37℃ for 25 min during which vibrated the suspensions discontinuously, once every 5 min. The cell suspension (20 μl) was mixed with low melting‐point agarose (200 μl) and thereafter spreading this mixture on a slide which was precoated with standard melting‐point agarose. After kept at 4℃, these slides were placed in lysing solution overnight. Then, the slides were placed in an electrophoresis chamber, which was filled with alkaline electrophoresis buffer allowing for 30 min to unwind the DNA. DNA electrophoresis was undertaken at 25 V for 30 min at 4℃. The reaction mixture was then neutralized and washed three times, with 0.4 M Tris‐HCl (pH 7.5). Thereafter, staining was done by adding ethidium bromide solution on each slide, and patterns of DNA fragment in each sample were observed through a fluorescence microscope (excitation 520 nm, emission 590 nm). 150 cells in each slide were observed to record olive tail moment (OTM) and tail DNA% (TD%) by the CASP software.

### 2.8 RNA collection and RT‐PCR analysis

Hippocampal tissue was dissected out and dissolved in TRIzol reagent. Total RNA was isolated and used to synthesize cDNA with the SYBR Premix Ex Taq II Reagent Kit and gDNA Eraser according to the manufacturer's instructions (TaKaRa, Japan). PCR cycles included initial denaturation at 95℃ for 5 min, and 40 cycles of denaturation at 95℃ for 15 s, annealing at 60℃ for 60 s and extension at 72℃ for 30 s. The reactions were conducted on the ABI PRISM 7,500 real‐time PCR system (Applied Biosystems, USA). The relative mRNA expression level was calculated using the comparative 2^–△△CT^ method and normalized by that of β‐actin. The primers (synthesized by Invitrogen Company) used for RT‐PCR were as followed. (Primer pairs used in RT‐PCR: BDNF: F:5'‐GGACATATCCATGACCAGAAAGAAA‐3' and R:5'‐GCAACAAACCACAACATTATCGAG‐3'; PSD‐95: F:5'‐AAGCGGGAATATGAGATAGACG‐3' and R:5'‐ATAGAGGTGGCTGTTGTACTGG‐3').

### 2.9 Western blotting analysis

Hippocampal tissue was lysed in ice‐cold radioimmunoprecipitation assay (RIPA) lysis buffer (Beyotime Biotechnology, Shanghai, China), and then centrifuged at 1,000 rpm for 10 min at 4℃. Equivalent amounts of protein (80 μg) were separated by 10% SDS polyacrylamide gel electrophoresis and transferred to PVDF membranes. The membranes were blocked with 5% nonfat milk (dissolved in triphosphate‐buffered saline with 0.1% Tween (TBST)) for 45 min at room temperature and incubated with primary rabbit antibodies for BDNF (1:800, immunoway), CREB1 (1:1,000, immunoway), p‐CREB1 (1:1,000, immunoway), Synapsin Ⅰ (1:800, abcam), p‐Synapsin Ⅰ (1:800, abcam), PSD‐95 (1:1,500, immunoway), and β‐actin (1:1,000, immunoway), respectively, diluted in blocking buffer at 4℃ overnight. After the membranes were then washed three times in TBST, the horseradish‐peroxidase‐conjugated goat‐anti‐rabbit IgG antibody (1:5,000, abcam) was added to allow incubation for 1 hr at room temperature. The membranes were washed three times in TBST, and the immunoblots were revealed with the chemiluminescent ECL Western Blotting Detection Reagent (Beyotime Biotechnology, China) and visualized by a chemiluminescence system (Tanon 5,200). Optical densities of the bands were scanned and quantified with ImageJ software.

#### 2.9.1 Statistical analyses

All values are expressed as means ± *SD*. We analyzed ELISA data of BDNF and neuroinflammation factors, mRNA expression levels in the hippocampus, band intensities, and other data with a one‐way ANOVA comparison test. Bonferroni's multiple comparison test was used for pairwise comparisons. Statistical analysis was performed using SPSS 23.0. The differences were considered statistically significant at *p < .05.

## 3 RESULT

### 3.1 General toxicity study

Either dams or pups in all groups gained weight steadily, and there were no visible changes in body weight or food and water consumption after the treatment period.

### 3.2 Effects of DCA on behavior

#### 3.2.1 Morris maze test

**FIGURE 1:**
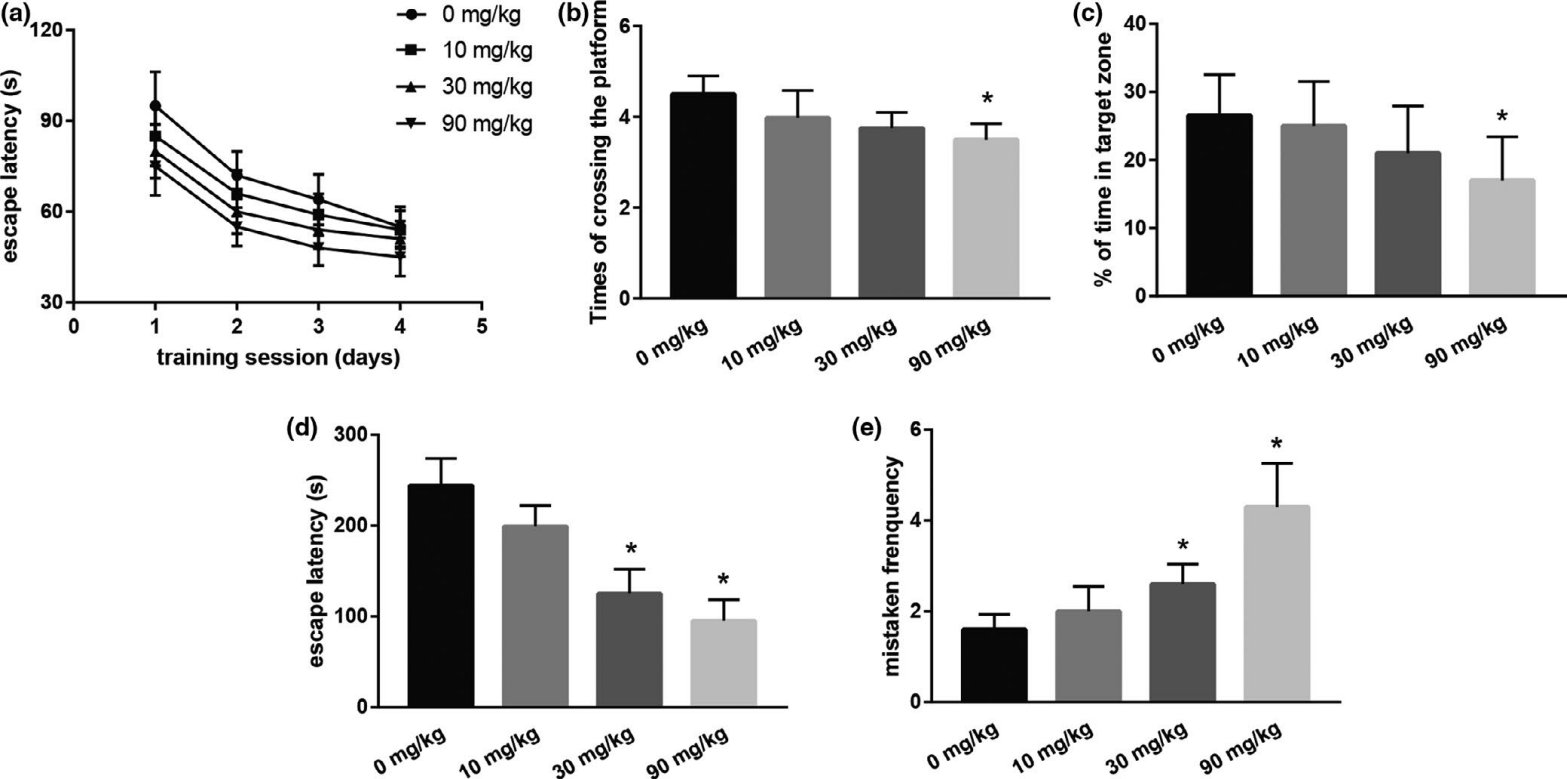
(a) Escape latency of the rats reaching the terrace in the morris water maze test during the training trial. Eight rats per group. (b) The times of rats passing the platform site. Eight rats per group. (c) Memory retention in probe trial test was expressed as the percentage of time spent in the target zone. Ten rats per group. (d) Escape latency of animals in the step‐down test. (e) Mistaken responses of animals in the step‐down test. Ten rats per group. Each column represents the mean ± *SD*; *p < .05 compared with the control

As shown in Figure 1a–c, escape latency, times of crossing the platform site, and time in target zone have been exhibited. The escape latency in the training trial is shown in Figure 1a analyzing by two‐way ANOVA, which showed significant differences among groups (group effect: *F* = 33.589, *p* < .05; training day effect: *F* = 358.096, *p* < .05, respectively). And during the training trial, in the 90 mg/kg group, the times of pups crossing the platform site was significantly lower than the control (*F* = 4.186, *p* < .05; Figure 1b). Besides, the analysis of the time in the target zone showed significant differences among groups during the probe test (*F* = 3.145, *p* < .05, Figure 1c). In the 90 mg/kg group, the time in the target zone was significantly lower in comparison with the control group (*p* < .05).

#### 3.2.2 Step down test

The results for memory latency were shown in Figure 1d. DCA treatment decreased the time taken to return to the bars. The times taken by the pups to return to the steel bars were significantly shorter in the 30 and 90 mg/kg group than the control (*p* < .05). Statistical analysis of mistaken responses is shown in Figure 1e, with results indicating that animals in the 30 and 90 mg/kg group made significantly more mistakes than those in the control group (*p* < .05).

### 3.3 Effects of DCA on hippocampal neuron structure and ultrastructure

#### 3.3.1 Optical microscopy

**FIGURE 2:**
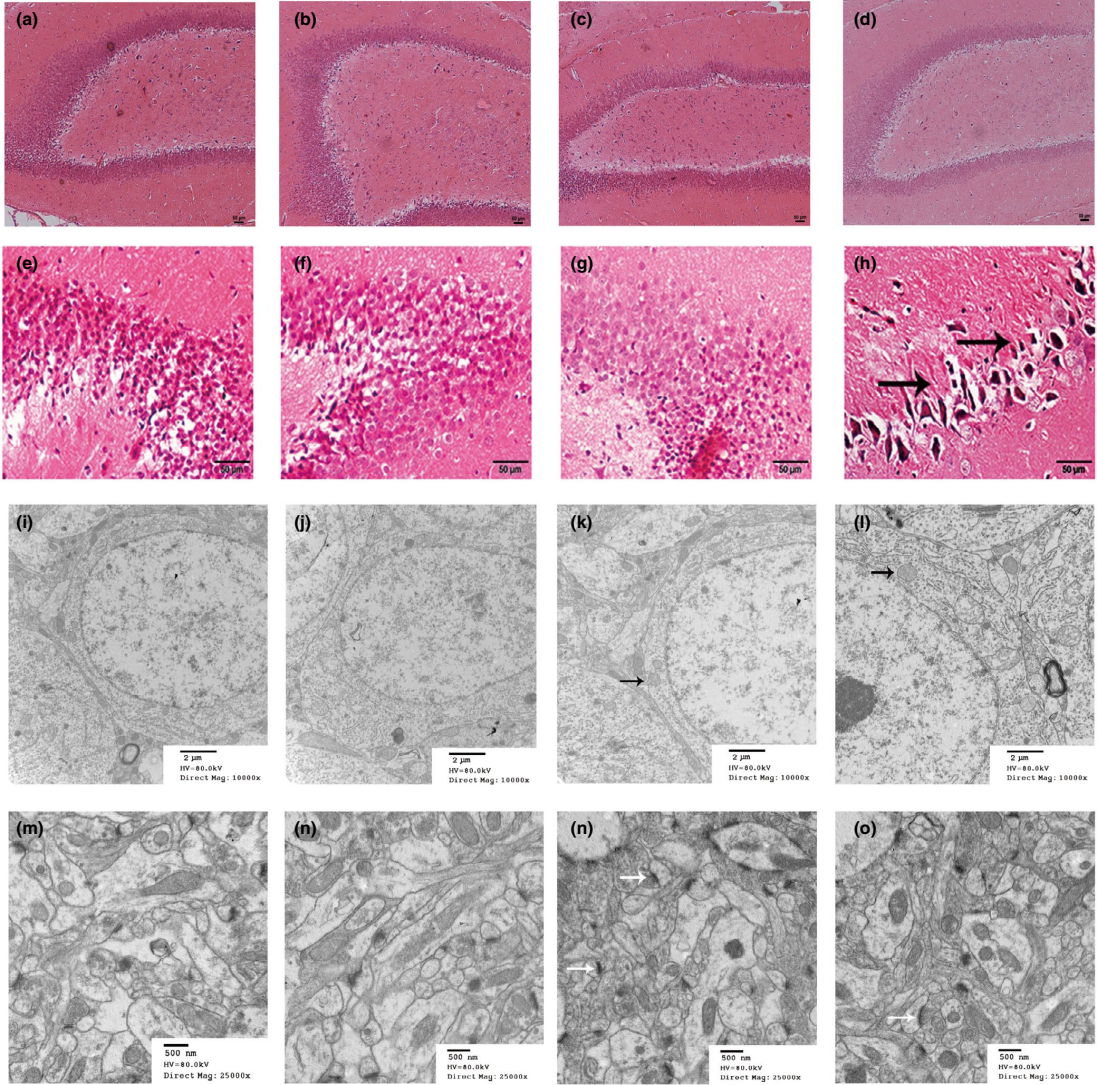
(a–h) Histopathological changes in rat hippocampi treated with DCA. Paraffin‐embedded rat hippocampus sections were stained with hematoxylin and eosin. In 0 mg/kg group: (a) (40×), e (400×); in 10 mg/kg group: (b) (40×), f (400×); in 30 mg/kg group: (c) (40×), (g) (400×); and in 90 mg/kg group: (d) (40×), (h) (400×) showed a sparse and irregular arrangement of neurons with shrunken nuclei (black arrows). (i–p) Effects of DCA on the microstructure of neurons under electron microscopy: in 0 mg/kg group: (i) (10,000×), (m) (25,000×); in 10 mg/kg group: (j) (10,000×), (n) (25,000×); and in 30 mg/kg group: (k) (10,000×), (o) (25,000×); in 90 mg/kg group: (l) (10,000×), (p) (25,000×). Swollen mitochondria, enlarged endoplasmic reticulum, and fuzzy nuclear membrane are marked with black arrows. Fuzzy structure of presynaptic, postsynaptic membranes and synaptic cleft are marked with white arrows

The results of hematoxylin–eosin staining showed morphological changes occurring in the hippocampi of the DCA‐treated groups (Figure 2a–h). In the control group, the neurons showed a compact and regular arrangement accompanying a large and round nucleus in each neuron. In the DCA‐treated groups, however, the number of cell layers was significantly decreased, and the neurons showed an irregular and sparse arrangement of cells with shrunken nuclei, especially in the 90 mg/kg group.

#### 3.3.2 Electron microscopy

Transmission electron microscopy showed that ultrastructural organizations of the hippocampi were changed in the DCA treatment groups. In control hippocampi, the neurons had smooth bilayer karyolemma and abundant cytoplasm containing healthy mitochondria with obvious cristae and endoplasmic reticulum with ribosomes (Figure 2i). However, in the DCA treatment groups (especially the 90 mg/kg group), circumferences of neurons were blurred, and karyolemma of the neurons were shriveled, the mitochondria were swollen with no cristae, and the endoplasmic reticulum was flattened with outspread saccules (Figure 2j–l). In the control group, presynaptic structures were clearly seen (Figure 2m). However, in the DCA treatment groups (especially the 90 mg/kg group), the formation of the synapse was damaged, which indicated the treatment of DCA could significantly aggravate the damage (Figure 2n–p). The arrows showed the structure of the presynaptic and postsynaptic membranes and the synaptic cleft.

### 3.4 Effects of DCA on oxidative stress‐associated indicators, neuroinflammation factors and BDNF level in the hippocampus

**FIGURE 3:**
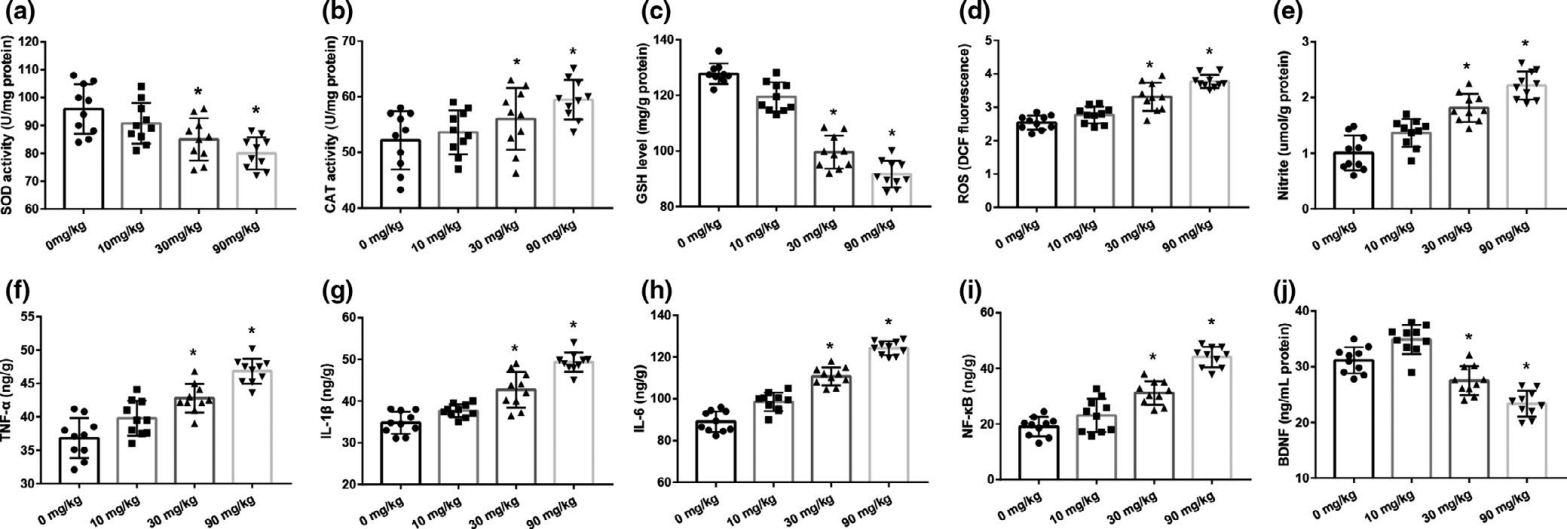
(a–e) Effect of dichloroacetic acid (DCA) on the activity of superoxide dismutase, catalase, and glutathione level and reactive oxygen species, nitric oxide contents in the hippocampus of the rats. (f–i) Effect of DCA on the neuroinflammation factors in the hippocampus of the rats. (j) Level of BDNF in the hippocampus was determined with an ELISA. Ten rats per group. Each column represents the mean ± *SD*; *p < .05 compared with the control

Figure 3a–e showed that the SOD, CAT, and GSH levels and ROS, NO content in the hippocampus of pups. DCA significantly inhibited the levels of SOD and GSH, while enhanced the CAT activity compared with the control group (*p* < .05). With the elevated doses of DCA, ROS and NO contents were significantly increased in a dose‐dependent manner. As is shown in Figure 3f–i, remarkable increases in TNF‐α, IL‐1β, IL‐6, and NF‐κB in DCA‐treated groups were found as a result of the modulation of inflammation response. The level of BDNF in the hippocampus was shown in Figure 3j. It was significantly decreased by 12.49% and 18.48% (*p* < .05) in the 30 and 90 mg/kg group respectively as compared with the control. However, compared with the control, it was slightly increased in the 10 mg/kg group, although the difference between the two groups was not statistically significant.

### 3.5 Effect of DCA on DNA damage in the hippocampus

**FIGURE 4:**
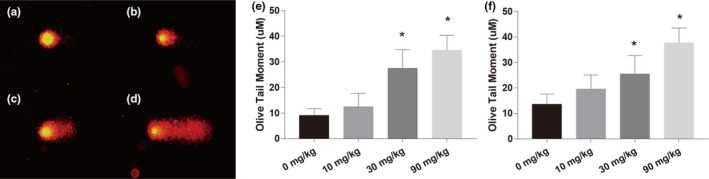
(a–d) Representative comet images of the control group and dichloroacetic acid (DCA) treatment groups. (e–f) Effects of DCA treatment on tail DNA% and olive tail moment. Eight rats per group. Each column represents the mean ± *SD*; *p < .05 compared with the control

The effect of DCA on DNA damage in the hippocampus of pups estimated by comet assay. As shown in Figure 4, it was observed that a normal cell comprised a single head (Figure 4a) while the cell in DCA‐treated group had both a head and a long tail. With the increase in dose, moreover, the degree of tailing and the fluorescence intensity in the tail of comet had significant changes (Figure 4b–d). The extent of DNA damage was estimated by determining OTM and TD%. OTM was significantly increased by 66.68% and 74.69%, and TD% was significantly increased by 43.87% and 66.88% (*p* < .05) in the 30 and 90 mg/kg group respectively as compared to the control. The data showed that the parameters like OTM (Figure 4e) and TD% (Figure 4f) were found to be significantly (*p* < .05) increased in DCA‐treated group compared with the control.

### 3.6 Effects of DCA on mRNA and protein expression

**FIGURE 5:**
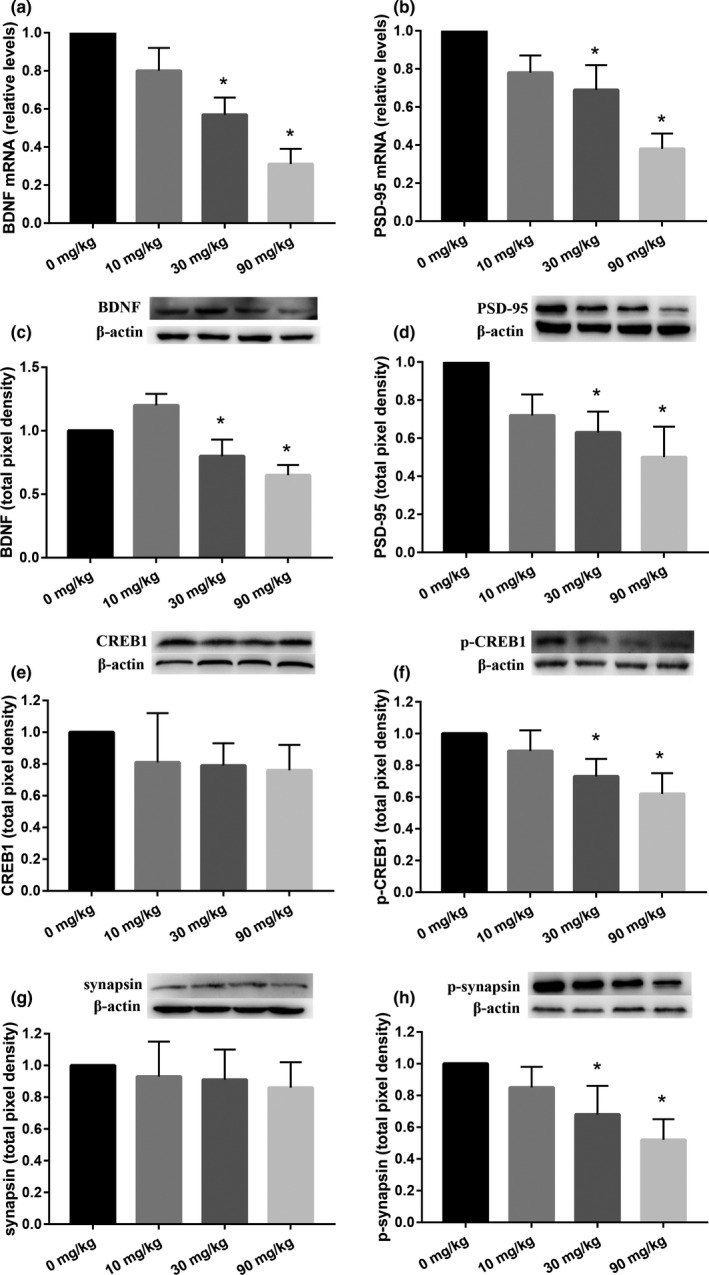
BDNF(5a) and PSD‐95(5b) mRNA expressions in the hippocampus were determined with RT‐PCR; Representative Western blots of BDNF (5c), PSD‐95 (5d), CREB1 (5e), p‐CREB1 (5f), synapsin Ⅰ (5g), p‐synapsin Ⅰ (5h) proteins were shown, together with the pixel densities. Eight rats per group. Each column represents the mean ± *SD*; *p < .05 compared with the control

The relative expression levels of BDNF, PSD‐95, and β‐actin mRNA were detected by real‐time RT‐PCR. 30 and 90 mg/kg groups had a lower level of BDNF mRNA (39.8%, 68.4%) and PSD‐95 mRNA (30.9%, 59.6%) expression compared with the control, respectively (Figure 5a,b). The effects of DCA on protein expression were evaluated with Western blotting, and the bands of them and the statistical analysis results were shown in Figure 5c–h (BDNF, PSD‐95, CREB1, p‐CREB1, synapsin I, and p‐synapsin I). Compared with the control group, the protein expression levels of BDNF, PSD‐95, p‐CREB1, and p‐synapsin I in the 30 and 90 mg/kg groups significantly decreased. However, significant changes in the protein expression levels of CREB1 and synapsin I were not observed in treatment groups. And a significant difference was also found in the ratio of CREB1 and p‐CREB1, synapsin I and p‐synapsin I which meant that DCA affected the production of p‐CREB1 and p‐synapsin I via weaken phosphorylation, not the reduction of total unphosphorylated CREB1 and synapsin I. However, there were no significant alterations on protein expression levels within the hippocampus in the 10 mg/kg group compared with the control, although a trend toward increasing level was evident.

## 4 DISCUSSION

In the present study, we novelly found that fetal exposure to DCA could hamper synaptic plasticity and cognition function of rats in adulthood. Oxidative stress and neuroinflammation, as well as an impaired neurotrophic factor and its downstream effectors such as synapsin Ⅰ, PSD‐95 and CREB1, could aggravate the deleterious effects of DCA on synaptic plasticity and cognitive function.

Moreover, taking into account the relatively low‐dose level that people exposed in daily life, our study reduced the dose of DCA and extended the exposure time comparing previous research (Delinsky et al., 2005). As we all know, the hippocampus is considered to be an essential region of the brain, closely associated with cognitive, learning, and memory functions (Rao, Mott, Wang, Chung, & Pak, 2013). Hippocampal oxidative stress could trigger the overproduction of mitochondria‐free radicals and deplete the antioxidant enzymes (Hafez et al., 2014). Excessive levels of ROS formed by the partial reduction of molecular oxygen could damage cells and genetic structures (Zhong et al., 2016). The present outcomes indicated that DCA exposure prompted the generation of ROS consistent with the results of the previous study (Pandey et al., 2014). NO and H_2_O_2_ are primary sources of ROS overproduction under different stress conditions (Funato, Michiue, Asashima, & Miki, 2006). Our study also demonstrated the content of NO and H_2_O_2_ significantly increased after fetal exposure to DCA, which has never been reported in previous studies. SOD could eliminate harmful substances produced by the body during metabolism and CAT and GSH act as an important antioxidant and free radical scavenger in the body via combining with free radicals and heavy metals (Molehin, Adeyanju, Adefegha, Oyeyemi, & Idowu, 2019). In the present report, fetal exposure to DCA significantly inhibited the activity of SOD and GSH contents and enhanced CAT activity in the hippocampus, which indicated that the mechanism of neurotoxicity in the hippocampus might be associated with oxidative damage.

Activated microglia could promote the generation of proinflammation factors through the revitalize of NF‐κB. Moreover, the production of NF‐κB, TNF‐α, IL‐6, and IL‐1β in the hippocampus were determined with the induction of inflammation response. Chemical irritates could induce NF‐κB activation via a mechanism involving ROS (Soares et al., 2017). In this study, we observed that DCA increased the expression of pro‐inflammatory cytokines NF‐κB, TNF‐α, IL‐6, and IL‐1β, which elucidated that DCA could induce inflammation responses in the hippocampus.

Several studies have shown that free radicals and inflammation factors could induce DNA damage and impair DNA repair system (Gaharwar, Meena, & Rajamani, 2017; Rashid, Chowdhury, Ghosh, & Sil, 2017; Soares et al., 2017), which in turn may cause the reduction of mRNA in BDNF and its downstream products (Koh et al., 2017; Wu et al., 2004). In our experiment, we identified evidence of DNA impairment in the hippocampus exposed to medium and high concentrations of DCA. In comet assays, the percentage of tail DNA in experimental groups changed according to the level of DCA, and the olive tail moment was also a characteristic of DNA damage.

Miletic et al. have shown that CREB1 acts as a crucial transcription factor and prompts the production of BDNF proteins, which are essential in neurodevelopment and neurogenesis in the hippocampus (Miletic, Pankratz, & Miletic, 2002). Besides, consistent with prior studies, treatment by DCA was found to promote the decline in SOD and CAT function in the hippocampus. Moreover, oxidative damage could affect various genes such as CREB1 and BDNF, associated with neuronal regeneration, depression, and cognition (Koh et al., 2017). It is well known that BDNF facilitates synaptic transmission by regulating synapsin Ⅰ phosphorylation (Wu et al., 2004), which plays a vital role in the maintenance of presynaptic structure and modulation of transmitter formation (Jovanovic, Czernik, Fienberg, Greengard, & Sihra, 2000). PSD‐95 scaffolding protein has been known as a marker of synaptic strength (Ehrlich, Klein, Rumpel, & Malinow, 2007), which could be regulated by BDNF via PI3K/Akt signaling (Li, You, Li, Qiu, & Wang, 2019), and it has been identified as a possible mechanism for synaptic plasticity and LTP. In the present report, the results showed that DCA damaged cognitive function and synaptic plasticity, and it seems to be associated with lower levels of BDNF and of its downstream effectors p‐synapsin I, p‐CREB1, and PSD‐95.

Interestingly, the result of ELISA on the hippocampal BDNF level showed that the amount of BDNF in the 10 mg/kg group increased slightly, which was in accordance with the results of BDNF protein, and we speculated the cause of this phenomenon might be hormesis of DCA. However, the mRNA of BDNF in the 10 mg/kg group decreased slightly compared with the control, although the difference had no statistical significance, and we conjectured the fact might be connected with a kind of microRNA or epigenetics such as DNA methylation which spurs further investigations. At all events, it is possible that fetal exposure to DCA may reduce BDNF‐related synaptic plasticity and cognitive abilities. Moreover, the oxidative stress, neuroinflammation response, and reduced neurotrophic factors should not be considered separately, which should be inversely viewed as an array of interconnecting signals, as much as to say that oxidative stress and neuroinflammation may aggravate the degree of decrease of neurotrophic factors.

Besides, the results of the behavioral experiments in this study are in agreement with the previous research (Moser et al., 1999), whereby the time in the target zone was reduced, and the latency was prolonged in the DCA‐treated groups. Moreover, histomorphological changes were readily detected in the treatment groups, especially under electron microscopy, which were probably caused by the relatively high dose of DCA, which may suggest that fetal exposure to DCA may exert adverse effects on behavior, cognition, and memory in the rat.

## 5 CONCLUSIONS

Collectively, we have shown that fetal exposure to DCA may have deteriorated effects on learning ability and cognitive and memory functions in rats in adulthood. Also, we provided evidence that fetal exposure to DCA could significantly elevate levels of oxidative stress and neuroinflammation, and compromise neurotrophic factors, thus damaging synaptic plasticity. The present research established a basis for further studies of the intrinsic molecular mechanisms underlying the neurotoxicity of DCA, which would promisingly spur further investigations to develop methods for avoiding DCA exposure during pregnancy.

## CONFLICT OF INTEREST

The authors declare that they have no conflict of interest.

## AUTHORS' CONTRIBUTION

**Yue Wang and Wenbo Jiang**: Investigation, Visualization, Writing—original draft, and Writing—review and editing. **Yue Wang and Qiuying Dong**: Data curation, Formal analysis, and Writing—review and editing. **Yue Zhao and Yingying Chen**: Data curation and Formal analysis.** Guoli Sun and Changhao Sun**: Funding acquisition, Project administration, Supervision, and Validation.

### Peer Review

The peer review history for this article is available at https://publons.com/publon/10.1002/brb3.1801.

## Data Availability

All data used during the study are available from the corresponding author by request.
